# Analysis of Urban Forest Healing Program Expected Values, Needs, and Preferred Components in Urban Forest Visitors with Diseases: A Pilot Survey

**DOI:** 10.3390/ijerph19010513

**Published:** 2022-01-04

**Authors:** Kwang-Hi Park

**Affiliations:** Department of Nursing, College of Nursing, Gachon University, Incheon 21936, Korea; parkkh@gachon.ac.kr; Tel.: +82-32-820-4204

**Keywords:** urban forest, disease, healing effect, survey

## Abstract

Although the effectiveness of urban forest therapy has been studied and proven, most people are not well aware of the positive healing effects of urban forests that are easily accessible in daily life compared to the known healing effect of forests located outside urban areas. In addition, there has been a study on the analysis of urban forest healing program needs in the general population, but there is a lack of evidence on the expected values and needs of urban forest healing for people with diseases. Therefore, this pilot survey aimed to investigate the expected values, needs, and preferred components of urban forest healing programs in urban forest visitors with disease via an online user survey and see if there were any differences in the purpose of the urban forest visits and expected values of urban forest healing depending on the type of disease. The survey was conducted on 294 urban forest visitors with diseases. About 79% of respondents agreed with the healing effects of urban forest, however most respondents expected healing effects on mental health rather than on physical health (“mood change” was the highest with score of 4.43/5, followed by “reliving stress” (4.35/5) and “mental and physical stability” (4.31/5)). In addition, more than 82.0% of respondents agreed to participate in the program if a healing program for disease was developed. The results of the current pilot survey indicate that the purpose of the urban forest visits and expected values of urban forest healing were largely not different by the type of disease, and people with disease had a relatively lower awareness and lower expected values of urban forest healing effects on physical health, but high demand for the program. Urban forest therapy programs should be developed based on the specific clinical characteristics of the disease to maximize the effectiveness of the program. Additionally, policies should be implemented to promote the beneficial effects of urban forest healing not only for mental health but also for physical health.

## 1. Introduction

Forest healing, which is a practice comprising of activities utilizing forests to improve immunity, mental health and physical health has been established as a culture in South Korea, with annual visits to forests exceeding one million in 2014 [1]. The number of visitors to forest healing centers has rapidly increased over time to reach 76,000 in 2010, 1.15 million in 2014, and 2.27 million 2019 in South Korea. Similarly the number of users of forest healing programs in the country has surged to 1067 in 2009, 1.7 million in 2015, and 1.8 million in 2019 [1]. In addition, it is predicted that the demand for forest healing will continue to increase with time due to the escalation in environmental health related risk factors, such as particulate matter in the air, as well as increased economic development and demand for leisure [2,3,4]. However, most of the healing forests are located in the suburbs, making it difficult to obtain healing benefits of forests in everyday life. In South Korea, most of the national and public healing forests are located on average 90 min away from metropolitan cities, by car. Moreover, it is particularly difficult for the mobility disabled, elderly, pregnant women, and those with diseases affecting movement, to use the healing forests due to the lack of accessibility [5]. In addition, the country has become an aging society with an aging rate of 7.2% in 2000. This rate increased to 14.3% in 2018, and is forecast to rise to 42.5% by 2065, making it the highest in the world [6]. As the national population continues to age, the interest in benefits of urban forests is also soaring. Therefore, interest in urban forests with high accessibility is increasing [7].

Urban forests were first defined by Jorgensen in 1974 as “a specialized branch of forestry, and it has in its objective the cultivation and management of trees for their present and potential contribution to the physiological, sociological, and economic well-being of urban society”. Thereafter, Deneke defined urban forestry in 1993 as “the sustained planning, planting, protection, maintenance, and care of trees, forests, greenspace, and related resources in and around cities and communities for economic, environmental, social, and public health benefits for people [7]”. Preserving forest cover as urban populations grow into surrounding rural areas, as well as trying to restore essential aspects of the urban environment after construction, are all part of the definition. Continued growth at the urban front increases environmental and public health concerns, as well as the possibility of generating educational and environmental links between nature and urban people. Urban and community forestry is composed of development of citizen engagement coupled with aid for investment in sustained tree planting, protection, and care programs. A multitude of definitions have been proposed over the years, but they all acknowledge that urban forests do not end at the limits of the city.

Nowadays, the main focus of primary health care practices is the identification of risk factors for preventing diseases and aims to improve the quality of life through this and the prevention of chronic conditions, which is different from the past that focused on diagnosing and treating diseases [8]. In particular, it is becoming important for most people living in cities to establish and implement long-term care plans to promote health as chronic diseases related to stress from their daily lives increase [9]. As such, the various therapeutic effects of urban forests have been proven. Especially in South Korea, where about 89% of the population lives in metropolitan areas, forest recreation and healing in urban forests that can be easily accessible by the people are needed [10].

Many previous studies have summarized the relationship between the natural environment and human health [11,12,13,14,15]. Recent trends indicate a growing interest in urban forests, as these are seen as a mechanism to encourage physical activity, facilitate social cohesion, and promote both psychological and physiological restoration [1,14]. Various studies have shown the benefits of urban forest therapy programs and their effects in a forest environment [11,12,15,16,17]. The Korean government is also promoting public health by designating the living areas and surrounding lands as urban forests for healing effect on stress caused by urban life [18]. Therefore, it is necessary to continuously develop various healing programs and related facilities for preventing disease and health in urban forests, and for this, the need for an evaluation of the multiple functions of urban forests, including healing benefits, is a prerequisite.

### 1.1. Operational Definition of “Urban Forest”

Referring to previous studies [19,20,21], in this study we defined urban forests as “Trees, forests, and greenspaces that are located within urban living area and play a plethora of ecological and social roles in the lives of local residents”. These are limited to places with an infrastructure for hiking trails and fitness facilities. Ecological functions include particulate matter reduction, noise reduction, furnishing animal and plant habitats, and providing green spaces, while social functions include exercise, rest, leisure, experience, and education.

### 1.2. Healing Effects of Forest

#### 1.2.1. General Benefits of Urban Forest

Urbanization, advancements in technology, crowding, and fast-paced life have dramatically decreased the time humans spend in natural surroundings. The lifestyle of people living in cities has been shown to produce negative emotions such as depression, pain and anxiety [22]. Ulrich, through his stress reduction theory, has described the need for urban residents to experience nature. This explains that interaction with nature helps in stress reduction as well as enhancing the physiological functioning of humans [11]. Forest healing or forest therapy is one of the therapies provided by nature for improving mental and physical health and includes many environmental factors such as landscape, phytoncides, sounds, lights, and negative ions [21]. Then, healing effect of urban green areas may provide feasible health benefits, as they are easily accessible [11]. Although it is relatively less than natural forests, the healing effects of urban forest have been proven in many studies [23,24,25]. As stated by Han et al., urban forests vary from one region to another, have several spaces and plantations as well as infrastructure for various forest activities, and therefore, they can be utilized by urban dwellers as a ‘healing space’ [17]. Social scientists have also established that urban forests and green spaces improve mental well-being [26].

Previous studies observed that forest healing programs in urban forests enhance mental health such as resilience, stress reduction [17,27]. Lee et al. concluded that the therapeutic effect of urban forest therapy on the psychological healing of middle-aged women thought focus group interview [11]. Furthermore, another study found that urban green space improved children’s emotional happiness and behavior resilience [28]. Urban forests have been found to be effective not only for mental health but also for physical health such as decreasing pulse rate, blood pressure, variability of heart rate, nervousness, tension, depression in middle-aged and elderly subjects [12,13,15,29]. A study on the association between urban tress and various health benefits revealed that more urban tree canopy were mainly associated with lower incidence of obesity, high blood pressure, asthma, and type 2 diabetes [30].

#### 1.2.2. Healing Effects of Forest Therapy for Chronic Diseases

Forest healing is not considered as a remedy for diseases, rather it’s a healing activity that aids in the maintenance of patients’ health and the enhancement of both physiological and psychological functions [1]. As such, many studies have been conducted that says that the healing effects of forest has a positive effect on various types of chronic diseases. Recent study have shown that forest therapy improves depression and anxiety in addition to reducing blood pressure by stabilizing the autonomic nervous system in elderly subjects with dementia [20]. Lee et al. examined the biophysical and psychosocial effects of different types of forest on middle-aged women with metabolic syndrome and found that wild forest had a positive effect on insulin responses, pulse rate, oxidative stress markers, and stress hormone level [31]. Chun et al. also demonstrated that forest therapy was beneficial for treating depression and anxiety symptom in patients with chronic stroke; therefore, forest therapy can be specifically used for chronic patients who cannot receive standard treatment [32]. In addition, many studies have shown that forest healing is effective in cardiopulmonary disease patients [33,34,35]. Direct evidence was provided by Mao et al. in favor of forest therapy being beneficial for patients with chronic heart failure and therefore it was considered that it has the potential to be used as an adjuvant therapy for cardiovascular disorders [34]. With regard to cancer patients, previous studies have stated that forest healing therapy not only increased physiological factors such as natural killer cell activity [36,37,38,39], but also psychological status such as depression, anxiety, and sleep quality [36,40,41,42].

### 1.3. Aim of the Study

Despite the studied and proven effectiveness of urban forest therapy, most people are not completely aware of the positive healing effects of urban forests compared to the healing effects of forests located outside urban areas.

Employing an Evidence-based practice (EBP) which is a problem-solving strategy that incorporates the best evidence of well-designed studies, experts’ opinion and patients’ value or preference is important to be established as a healthcare program which can lead to better patient outcome for individuals with diseases [43]. A lot of evidence already supports that forest healing program has a positive effect on improving physiological and psychological functions and many experts assured that the importance of forest healing program should be highlighted. However, although there has been a study on the analysis of urban forest healing program needs in the general population [44] there is still a lack of evidences to infer the needs and expected value of urban forest healing programs for individuals with diseases. Therefore, the purpose of this pilot survey was to investigate the purpose of the urban forest visits, expected values, and preferred components of urban forest healing programs based on developed questionnaires by expertise for urban forest visitors with known diseases in South Korea. The secondary purpose of the study was to see if there were any significant differences in the purpose of the urban forest visits and expected values of urban forest healing depending on the type of disease.

## 2. Materials and Methods

### 2.1. Ethical Approval

The study was conducted in accordance with the guidelines of the Declaration of Helsinki and approved by the Gachon University Institutional Review Board (1044396-202106-HR-136-01). The purpose of this study was fully explained before the survey was conducted. All participants who agreed to participate in the survey were required to sign an informed consent form before beginning the survey.

### 2.2. Participants

A total of 294 urban forest visitors who had visited urban forests within the last two years with disease (aged between 15 and 69 years), residing in two metropolitan cities in South Korea participated in an online survey conducted by a research institution with a panel composed of the same distribution as the Korean population sensor and secure representation. A non-probability sample extraction was used to select 294 subjects with diseases classified in the Korea National Health and Nutrition Examination Survey in 2019. Three thousand seven hundred thirty-five emails were distributed to the panel, 1239 checked their emails and 407 participated in the survey. Among them, 15 respondents who did not meet the inclusion criterion and 98 respondents who did not complete the survey were excluded, then, a total of 294 were included as a valid sample for this pilot survey. The respond rate was 32.8%.

### 2.3. Questionnaire

A questionnaire was created for this study in order to investigate the awareness of urban forest visitors with regard to the effects of urban forest healing. A survey titled “Questionnaire on expected values and needs of urban forest healing effects” was developed through systematic literature reviews of previous studies [19,44,45] and consultation with a panel of experts. A draft questionnaire was prepared after several meetings with reference to the literature review. Next, the validity of the draft questionnaire was investigated by a panel of experts to determine whether it included appropriate components for the purpose of the study and was suitable for the patients. The questionnaire consisted of four sections: (1) general characteristics of the respondents, (2) purpose of urban forest visit, (3) expected values of the urban forest healing effect, (4) needs of urban forest healing programs, and (5) preferred components of urban forest healing programs. General population characteristics included age, sex, education level, marital status, income, occupation, and presence of disease. The purpose of the urban forest visits, expectation values of the urban forest, and needs of the urban forest healing program were rated on a 5-point Likert scale.

### 2.4. Data Collection

An online survey was conducted between 21 to 29 July 2021. The questionnaire was distributed to residents of two metropolitan cities with urban forests in South Korea via email with an online survey link. The purpose of the study was described in the first part of the questionnaire, and all valid questionnaires collected during the survey period were used for the analysis.

### 2.5. Statistical Analysis

The data were analyzed using the statistical software IBM SPSS Statistics for Windows, version 26.0 (IBM Corp., Armonk, NY, USA). Frequency analysis was performed according to the characteristics of each item. Quantitative variables were calculated as mean and standard deviation (SD), while categorical variables were presented as frequency and percentage. The Kolmogorov-Smirnov test was used to test for the normality of the data.

A chi-squared test was used to compare the distribution of preferred components of urban forest healing program in the age and disease categories. One-way repeated analysis of variance (ANOVA) was used to compare the expected values among type of disease. Additionally, one-way analysis of covariance (ANOCA) test with Bonferroni-adjusted post hoc test was used to explore the influences of any variables over the dependent variables. The level of significant was set at α = 0.05.

## 3. Results

Demographic characteristics of the respondents are described in Table 1. Among the respondents, 50.5% were women, and the average age was 50.3 ± 14.5 years. The most common primary diseases reported were circulatory system diseases (55.4%), followed by respiratory system diseases (11.6%), and musculoskeletal system diseases (9.5%). Multiple responses were available if there were more disease other than the primary disease, and 83 respondents had more than two diseases. Including multiple responses, a total of 294 respondents had 398 diseases. The most common diseases were circulatory system disease (43.7%), followed by endocrine system and metabolic diseases (17.1%), respiratory system diseases (12.6%), musculoskeletal system diseases (10.8%), depression (8.8%), cancers (5.0%), and others (2.5%). Other diseases included atopic dermatitis, Parkinson’s disease, and prostate disease.

### 3.1. Purpose of Urban Forest Visit

“Purpose of the urban forest visit” section of the questionnaire contained 18 sub-objectives under four major categories, all of which could be scored between 1 and 5 points (1 point: least, 5 points: best). Among the four major categories, “rest/healing” was the highest scored with 3.97 out of 5, followed by “nature-friendly” (3.70). “risk aversion” was the least common purpose of the urban forest visit (3.52). Among the sub-objectives, “taking a walk” under “healthcare” section had the highest score of 4.40 and “maintaining health” followed closely at 4.09. However, the overall score of “healthcare” was low because of the low scores of “healing a disease” (2.80) and “participating in healing program” (3.06) sub-objectives (Table 2).

The purpose of the urban forest visit was significantly different among type of disease in “healthcare” in the major category, as well as “healing program participation” and “maintaining health” under “healthcare” section (*p* < 0.05). However, there was no significant difference in the purpose of the urban forest visit after controlling for the effect of age (*p* > 0.05).

### 3.2. Expected Values of the Urban Forest Healing Effect

Table 3 shows expected values of the healing effect of urban forest. Before, asking the expected values of the urban forest healing, the respondents were asked whether they agreed with the fact that urban forests have healing effects. Two hundred thirty-four (79.6%) respondents agreed with the healing effects of urban forest. The agreement of the urban forest healing effect was significantly different depending on the type of disease (*p* < 0.001).

Only those who agreed were given the follow-up questionnaire. Among the healing effects provided by urban forest, “mood change” scored the highest with 4.43 out of 5, followed by “relieving stress” (4.35) and “mental and physical stability” (4.31). “Prevention of diseases” scored the lowest with 3.73 points followed by “Improving immunity” (3.93).

Expected value of the urban forest healing effect was significantly different among type of disease in “healthcare”, “improving immunity”, “prevention of diseases”, and “rejuvenation” (*p* < 0.05). However, only the expected values of “improving immunity” differed significantly on types of disease after controlling for the effect of age (F_(5227)_ = 2.208, *p* = 0.047). Bonferroni-adjusted post hoc test showed that respondents with circulatory system diseases had significantly higher expected value of the urban forest healing effect than those with endocrine system and metabolic diseases in terms of “improving immunity” (*p* = 0.035).

### 3.3. Needs of Urban Forest Healing Programs

Before proceeding with this part of the survey, the respondents were asked whether they needed an urban forest healing program. Of the total respondents, 81.3% agreed on the need for an urban forest healing program, and only 3.7% disagreed (Table 4).

When asked the question, “Would you like to participate in the program if healing program is established in an urban forest for diseases?” 82.0% respondents “agreed” to participate, while 5.4% did not want to participate in the program.

The necessity and the intention to participate in the urban forest healing program participation were not significantly different depending on the type of disease (*p* > 0.05).

The most appropriate time duration of the healing program was 60 min for 121 respondents (50.2%), followed by 120 min for 51 patients (21.2%), and 90 min for 38 respondents (15.8%). Among the respondents, 106 (44.0%) thought that the appropriate cost of the healing program in urban forest per visit was “less than KRW 5000 (USD 4.2)” and 78 (32.4%) thought it should be within “KRW 10,000 (USD 8.4)” (Table 4).

### 3.4. Preferred Components of Urban Forest Healing Program

Table 5 shows the preferred components of the urban forest healing program for urban forest visitors with disease. This part of questionnaire was required to be completed by the respondents who were willing to participate in the program. Multiple responses were provided. The most preferred component of the urban forest healing program as reported by the respondents was, “taking a walk” (73.8%). “meditation” (57.7%) and “wind bath” (50.2%), were also selected as components of high preference by 57.7% and 50.2% of the respondents, respectively. On the other hand, only 23.1% agreed to inclusion of “art therapy” in the program; “forest recreation” (24.9%) and “tea ceremony” (31.1%), were selected as components that were less preferred.

## 4. Discussion

This pilot survey aimed to investigate the expected values, needs, and preferred components of urban forest healing programs in urban forest visitors with disease and see if there were any significant differences depending on the type of disease through a user survey. Respondents were classified on the basis of disease types, and circulatory system disease was reported by 55.4%, respiratory system diseases by 11.6%, and musculoskeletal system diseases by 9.5% of the respondents in our study. About 70% of the participants had diseases that demanded lifestyle improvements, including exercise and diet modifications. This is confluent with the recent observations that people with various types of disease are visiting urban forests and indicates that the need as well as demand for urban forest healing programs is expected to rise in the future.

In the present study the most common purpose of visit to the urban forest was reported as “resting and healing” followed by “nature-friendly” and “healthcare”. The “taking a walk” sub-objective of the “healthcare” section scored high but “prevention of disease” and “healing a disease” scored relatively low. These results are similar with the findings of the previous study on the use of urban forests by visitors with or without diseases, showing “change of mood” and “mental health promotion” as the highest stated purposes of urban forest visits with “healthcare” being the least common reported purpose [44]. The purposes of visit to the urban forest were not significantly different by type of disease after controlling ‘age’. This shows that urban forest visitors with the purpose of “healthcare” are more affected by age than type of diseases.

Many studies on forest healing effects have consistently shown psychological benefits such as reduction in depression and anxiety, as well as physical effects such as decreased blood pressure and increased NK cell activity [1,11,13,40,46]. However, people without disease and even with disease were only aware of the psychological effects of forest healing and were relatively unaware of the effects of forest healing on physical aspects of diseases.

In the current study 79.6% of the respondents agreed on the healing effect of urban forest, which is similar to the results of a survey in Germany where 77.2% of respondents were positive of the healing effect of urban forests, of which 45.5% were very confident about the healing effect [10].

However, it was seen that the agreement with regard to physical healing effects such as “improving immunity”, and “prevention of disease” was relatively low compared to psychological healing effects such as ‘mood change’ and “reliving stress”. This shows that the expectation of physical healing related to urban forest visits is low among people, which is also reflected in the purpose of urban forest visits. This is in a similar to the previous study, which reported that the artificial landscape beauty of urban forests is relatively less evaluated for the healing function of forests than ecologically well-cultivated forests [9]. In the result of the present survey, the expected values of “improving immunity” significantly higher in patients with cardiovascular system disease after controlling for the effect of age. This is believed to be due to the fact that urban forest visitors with cardiovascular disease expect more to promote immunity because the immune system plays an essential role in the development and progression of cardiovascular diseases [47].

About 81% of respondents agreed on the necessity of urban forest healing programs, and 82% of them were willing to participate in the program and it did not differ by the types of disease. The intention to participate in the programs was much higher in the current study than in previous surveys showing that 47.2% of the national population [48] and 58.3% of urban forest visitors [44] were willing to participate in the forest healing programs. However, as Park et al. [44] did not analyze the intention to participate in the urban forest healing program based on the presence or absence of disease, this study cannot be directly compared with our study. It can be assumed that the respondents included in the current study had a higher intention to participate in these programs because they had diseases. Applying the program in combination with the conventional treatment in people with diseases who are understood to have higher needs for forest healing programs could have the effect of reducing social and economic costs of diseases or other health related conditions on the healthcare system.

The preferred activities for urban forest healing programs in the decreasing order were walking, meditation, wind bathing, and forest gymnastics. These results are somewhat different from the previous studies on forest healing program preferences, where forest gymnastics, wind bathing, and meditation were the highest rated activities [49,50]. ‘Walking’ was the highest preferred activity as part of the healing program in the present study and the biggest purpose of urban forest visits, but the expected effect on physical health was relatively low. This might be due to a relatively lack of understanding of the fact that walking in the forest has a positive impact on physical health. In general, people are well aware of the physical benefits of ‘walking’, but the connection to physical fitness seems to be relatively insignificant because they think that ‘walking’ in the forest has greater impact on psychological aspect than ‘walking’ in the concrete. This is supported by a previous study analyzing big data on forest walking showed that walking in the forest tends to be perceived as leisure activities that taking a walk in the forest [51]. In addition, our results indicated that visitors with diseases had higher demands for physical activities such as ‘walking’ and ‘gymnastics’ than people without diseases, as part of urban forest healing programs, and this should be considered when developing suitable urban forest healing programs for people with diseases. Moreover, since it is important to constantly participate in these healing programs at places which are easily accessible in daily life, urban forests may be more suitable in terms of use for people with diseases than forests located outside urban areas. Furthermore, it was noted that except ‘walking’, the preference for physical health-related activities was low, and further studies should be conducted in order to promote the physical health benefits of urban forests, such as ’health promotion’ and ‘improving immunity’ in addition to the mental health benefits.

According to a Delphi survey conducted on forest healing by 19 experts in medicine, psychology, and forestry, to predict the preferred targets and diseases that can be applied to forest healing programs [52], the suitability was reported in the following order: moderate respiratory, endocrine, nutritional and metabolic, cardiovascular, and digestive diseases. Specifically, asthma, diabetes, obesity, high blood pressure, hyperlipidemia, myocardial infarction, and indigestion were identified as the target diseases. Expectations for forest healing effects and urban forest utilization can be promoted in daily life through efforts to clearly distinguish whether it is a competitive or complementary approach to the existing treatment methods.

In our study, about 81.6% participants responded that they would participate in the urban forest healing program for disease and considering the cost that these respondents were willing to pay for the program as per the results of the survey, it would be quite valuable to develop and publicize these healing programs. Correspondingly, the Korean government is actively making efforts to provide forest healing effects within living areas. At this point, it is very encouraging that awareness of the healing effect of urban forests is positive. Therefore, efforts will be needed to clearly present the effectiveness of forest healing programs by developing individualized healing programs for patients and expanding these to urban forests that are easily accessible in daily life.

To the best of our knowledge, this is the first study to investigate the expected values, needs, and preferred components of urban forest healing programs in urban forest visitors with diseases. However, the limitations of this study must also be acknowledged. First, the study was at risk of selection bias, as only those interested in the topic would have participated in the survey. Further research should use probability sampling methods to reduce this selection bias. Second, this pilot survey included only urban forest visitors with diseases; therefore, it is difficult to confirm whether our results were due to the presence or absence of diseases. Therefore, further studies are warranted to compare urban forest visitors with and without diseases to obtain meticulous results. In addition, the sample size of each disease was unequal so that type I error levels may not be guaranteed. Therefore, further research should include equal sample sizes considering age distribution for each disease to find out the difference depending on the type of disease and see their relationship to the degree of awareness or preference of urban forest healing effects, due to various clinical and pathological characteristics of these diseases. Future studies should focus on the detailed clinical characteristics of visitors to improve or develop customized urban forest healing programs for people with various types of diseases.

## 5. Conclusions

About 82% of urban forest visitors with disease agreed on the need for urban forest healing programs and wanted to participate in these programs. The results of the present survey showed that the purpose of the urban forest visits and expected values of urban forest healing were mostly not different for each disease and although people with diseases had relatively lower awareness and expected values of urban forest healing effects on physical health, the demand for these programs was still high.

Urban forest therapy programs should be developed based on the medical characteristics of the individual disease to maximize the effectiveness of the program. Additionally, policies should be made to inform general population that urban forest healing is beneficial not only for mental health but also for physical health. Moreover, our results can be used as basic data for the development of such programs for people with diseases.

## Figures and Tables

**Table 1:** Demographic characteristics of the respondents (*n* = 294).

Characteristics	Total (*n* = 294) *n* (%) or Mean ± SD
Age (year), mean ± SD	50.3 ± 14.5
Gender (women), *n* (%)	134 (45.6)
Education level, *n* (%)	
No education or elementary school or middle school	5 (1.7)
High school	82 (27.9)
Undergraduate	167 (56.8)
Graduate school	40 (13.6)
Marital status, *n* (%)	
Single	78 (26.5)
Married	206 (70.1)
Divorced	10 (3.4)
Economic status, *n* (%)	
Low (below KRW 3,000,000)	79 (26.9)
Middle (KRW 3,000,000–7,000,000)	156 (53.1)
High (above 7,000,000)	45 (15.3)
Missing	14 (4.8)
Type of primary disease, *n* (%)	
Circulatory system diseases	163 (55.4)
Musculoskeletal system diseases	28 (9.5)
Respiratory system diseases	34 (11.6)
Endocrine system and Metabolic diseases	27 (9.2)
Cancers	14 (4.8)
Depression	24 (8.2)
Others	4 (1.4)

**Table 2:** Questionnaire derived data on the purpose of visit to urban forest in urban forest visitors (*n* = 294).

Purpose of Visit to Urban Forest		Circulatory Diseases (*n* = 163)	Musculo-Skeletal Diseases (*n* = 28)	Respiratory Diseases (*n* = 34)	Endocrine & Metabolic Diseases (*n* = 27)	Cancers (*n* = 14)	Depression (*n* = 24)	Others (*n* = 4)	Overall	F(*p*)	F(*p*) ^†^
Rest /Healing	Rest/healing	4.16 ± 0.63	3.94 ± 0.96	4.19 ± 0.55	3.91 ± 0.88	3.90 ± 1.04	4.04 ± 0.81	3.39 ± 0.68	4.09 ± 0.72	1.549 (0.163)	-
	Spending time in nature	4.02 ± 0.75	4.16 ± 0.88	3.83 ± 0.89	4.07 ± 0.66	3.79 ± 1.22	3.89 ± 0.41	3.93 ± 0.41	3.99 ± 0.81	0.598 (0.732)	0.215 (0.972)
	Escaping from daily routine	3.84 ± 0.82	3.70 ± 1.09	3.64 ± 1.00	3.82 ± 0.93	3.74 ± 1.28	3.93 ± 0.88	2.86 ± 1.17	3.79 ± 0.91	1.034 (0.404)	-
	Relieving stress	3.99 ± 0.75	3.66 ± 0.76	3.79 ± 0.96	3.79 ± 1.00	4.01 ± 0.75	3.89 ± 0.88	3.57 ± 0.58	3.91 ± 0.81	0.944 (0.464)	-
	Physical/mental rejuvenation	4.15 ± 0.70	3.94 ± 0.71	4.02 ± 0.83	3.94 ± 0.96	3.96 ± 0.80	4.04 ± 0.84	3.75 ± 0.90	4.07 ± 0.76	0.671 (0.673)	-
	Overall	4.03 ± 0.56	3.88 ± 0.77	3.90 ± 0.65	3.91 ± 0.71	3.88 ± 0.70	3.96 ± 0.77	3.50 ± 0.59	3.97 ± 0.63	0.845 (0.536)	-
Healthcare	Taking a walk	4.42 ± 0.62	4.53 ± 0.51	4.45 ± 0.52	4.53 ± 0.63	4.12 ± 0.97	4.07 ± 0.70	4.46 ± 0.68	4.40 ± 1.27	1.725 (0.115)	1.379 (0.223)
	Healing program participation	3.24 ± 1.14	3.35 ± 1.25	2.38 ± 1.15	2.92 ± 1.05	3.13 ± 1.19	2.50 ± 1.32	3.39 ± 0.68	3.06 ± 0.64	3.463 (0.003)	1.300 (0.257)
	Simple exercise	3.87 ± 0.90	3.94 ± 0.74	3.83 ± 0.69	3.70 ± 0.90	3.30 ± 1.15	3.46 ± 1.04	3.75 ± 0.90	3.80 ± 1.19	1.496 (0.180)	-
	Maintaining health	4.20 ± 0.75	4.29 ± 0.61	3.90 ± 0.77	4.10 ± 0.58	3.90 ± 1.22	3.61 ± 1.05	3.39 ± 0.68	4.09 ± 0.80	2.915 (0.009)	1.152 (0.333)
	Prevention of disease	3.55 ± 0.96	3.63 ± 0.91	3.00 ± 1.08	3.54 ± 0.87	3.30 ± 1.22	3.21 ± 1.02	2.86 ± 1.17	3.45 ± 0.99	1.971(0.070)	0.607 (0.724)
	Healing a disease	2.87 ± 1.04	2.89 ± 0.85	2.40 ± 0.91	2.95 ± 1.02	2.91 ± 1.07	2.75 ± 1.07	2.14 ± 0.58	2.81 ± 1.01	1.332 (0.243)	-
	Overall	3.69 ± 0.65	3.77 ± 0.57	3.33 ± 0.56	3.62 ± 0.60	3.44 ± 0.69	3.27 ± 0.84	3.33 ± 0.51	3.60 ± 0.66	2.781 (0.012)	0.701 (0.649)
Risk Aversion	Avoiding particulate matter	3.02 ± 1.01	3.14 ± 0.93	2.69 ± 1.04	3.01 ± 0.78	2.86 ± 0.93	2.82 ± 0.91	2.14 ± 1.01	2.96 ± 0.98	1.159 (0.329)	0.330 (0.921)
	Breathing in fresh air	3.86 ± 0.88	4.01 ± 0.88	3.69 ± 1.11	3.79 ± 0.82	3.85 ± 1.07	3.68 ± 1.07	2.68 ± 1.58	3.81 ± 0.94	1.353 (0.234)	0.842 (0.538)
	Avoiding noise in the city	3.53 ± 0.94	3.57 ± 0.94	3.07 ± 1.27	3.70 ± 0.80	3.35 ± 1.03	3.61 ± 1.10	3.39 ± 1.22	3.49 ± 0.99	1.217 (0.298)	-
	Avoiding heat island phenom	3.80 ± 0.86	4.01 ± 0.85	3.64 ± 0.98	3.91 ± 0.83	3.74 ± 0.93	3.93 ± 0.91	3.39 ± 0.36	3.81 ± 0.87	0.659 (0.683)	-
	Overall	3.55 ± 0.78	3.68 ± 0.80	3.27 ± 0.94	3.60 ± 0.69	3.45 ± 0.89	3.51 ± 0.85	2.90 ± 0.40	3.52 ± 0.81	1.116 (0.353)	0.478 (0.824)
Nature Friendly	Enjoying the natural scenery	3.75 ± 0.87	3.73 ± 0.78	3.71 ± 1.03	3.70 ± 0.74	3.79 ± 0.68	3.89 ± 0.91	3.39 ± 0.68	3.74 ± 0.86	0.235 (0.965)	-
	Using nature and green space	3.89 ± 0.79	3.91 ± 0.77	3.95 ± 0.89	3.98 ± 0.64	3.96 ± 0.55	3.79 ± 0.77	3.93 ± 0.41	3.90 ± 0.76	0.151 (0.989)	-
	Communicating with nature	3.51 ± 0.96	3.42 ± 0.91	3.36 ± 1.03	3.42 ± 0.99	3.02 ± 1.34	3.61 ± 1.07	2.86 ± 1.54	3.45 ± 1.01	0.859 (0.526)	0.859 (0.526)
	Overall	3.70 ± 0.77	3.69 ± 0.71	3.67 ± 0.84	3.70 ± 0.62	3.59 ± 0.74	3.76 ± 0.76	3.39 ± 0.63	3.70 ± 0.75	0.197 (0.977)	0.308 (0.933)

^†^ After controlling the covariate (age) which was significantly related to the dependent variables.

**Table 3:** Expected values of urban forest healing effect in urban forest visitors who agreed with the urban forest healing effect (*n* = 234).

Expected Values of Urban Forest Healing Effect	Circulatory Diseases (*n* = 133)	Musculo-Skeletal Diseases (*n* = 26)	Respiratory Diseases (*n* = 31)	Endocrine & Metabolic Diseases (*n* = 22)	Cancers (*n* = 9)	Depression (*n* = 13)	Overall	F(*p*)	F(*p*) ^†^
Healthcare	4.38 ± 0.55	4.15 ± 0.54	4.03 ± 0.70	4.32 ± 0.60	4.44 ± 0.60	4.34 ± 0.62	4.30 ± 0.59	2.343 (0.042)	1.293 (0.268)
Increasing exercise effects	4.35 ± 0.57	4.18 ± 0.63	4.06 ± 0.62	4.16 ± 0.61	4.05 ± 0.80	4.29 ± 0.71	4.26 ± 0.61	1.771 (0.120)	-
Maintaining vitality	4.38 ± 0.53	4.09 ± 0.62	4.15 ± 0.54	4.29 ± 0.54	4.37 ± 0.56	4.23 ± 0.46	4.30 ± 0.55	1.932 (0.090)	1.318 (0.257)
Relieving stress	4.40 ± 0.50	4.37 ± 0.68	4.24 ± 0.52	4.32 ± 0.60	4.13 ± 0.69	4.34 ± 0.62	4.35 ± 0.55	0.770 (0.573)	-
Mental and physical stability	4.34 ± 0.55	4.23 ± 0.60	4.12 ± 0.66	4.38 ± 0.60	4.44 ± 0.48	4.45 ± 0.31	4.31 ± 0.56	1.192(.314)	-
Mood change	4.47 ± 0.50	4.23 ± 0.64	4.33 ± 0.58	4.48 ± 0.50	4.52 ± 0.51	4.40 ± 0.49	4.43 ± 0.53	1.253 (0.285)	-
Improving immunity	4.40 ± 0.63	3.90 ± 0.65	3.73 ± 0.84	3.60 ± 0.52 *	4.21 ± 0.56	4.07 ± 0.84	3.93 ± 0.68	2.328 (0.044)	2.208 (0.047)
Prevention of diseases	3.84 ± 0.74	3.63 ± 0.57	3.36 ± 0.96	3.57 ± 0.73	3.97 ± 0.81	3.79 ± 0.94	3.73 ± 0.79	2.399 (0.038)	1.359 (0.241)
Rejuvenation	4.31 ± 0.57	3.93 ± 0.84	3.96 ± 0.66	4.12 ± 0.73	4.29 ± 0.51	4.29 ± 0.51	4.20 ± 0.64	2.844 (0.016)	2.029 (0.062)

* Significant difference from those who with circulatory system diseases after controlling the covariate (age). ^†^ After controlling the covariate (age) which was significantly related to the dependent variables.

**Table 4:** Needs of urban forest healing programs with preferred duration and cost.

Items	Total (*n* = 294) *n* (%)
Necessity of urban forest healing program	
Strongly agree and agree	239 (81.3)
Strongly disagree and disagree	11 (3.7)
Neither agree nor disagree	44 (15.0)
Will you participate in urban forest healing program?	
Strongly agree, agree, somewhat agree	241 (82.0)
Strongly disagree, disagree, somewhat disagree	16 (5.4)
Neither agree nor disagree	37 (12.6)
Preferred duration of urban forest healing program *	
30-min	25 (10.4)
60-min	121 (50.2)
90-min	38 (15.8)
120-min	51 (21.2)
More than 120-min	6 (2.5)
Preferred cost of urban forest healing program *	
Free	1 (0.4)
Below KRW 5000 (USD 4.2)	106 (44.0)
KRW 10,000 (USD 8.4)	78 (32.4)
KRW 10,001—20,000 (USD 8.4—16.8)	50 (20.7)
Above KRW 20,000 (USD 16.8)	6 (2.5)

* Duration and cost of urban forest healing program were asked from only those respondents who were willing to participate in the urban forest healing program (*n* = 241).

**Table 5:** Preferred components of urban forest healing program.

Items	Total (*n* = 241) *n* (%)
Meditation	139 (57.7)
Taking a walk	178 (73.8)
Mental and physical reinforcement	117 (48.5)
Forest gymnastics	109 (45.2)
Forest recreation	60 (24.9)
Ecological experience	88 (36.5)
Wind bath	121 (50.2)
Performance in the forest	95 (39.4)
Tea ceremony	75 (31.1)
Art therapy	65 (22.1)
Aroma therapy	84 (34.9)

Multiple answers were available.

## Data Availability

The datasets generated during this study are available from the corresponding author upon reasonable request.
